# Identification, Characterization, and Expression Patterns of TCP Genes and microRNA319 in Cotton

**DOI:** 10.3390/ijms19113655

**Published:** 2018-11-20

**Authors:** Zujun Yin, Yan Li, Weidong Zhu, Xiaoqiong Fu, Xiulan Han, Junjuan Wang, Huan Lin, Wuwei Ye

**Affiliations:** 1 Research Base, Zhengzhou University, State Key Laboratory of Cotton Biology, Institute of Cotton Research of Chinese Academy of Agricultural Sciences, Anyang 455000, Henan, China; yinzujun@caas.cn (Z.Y.); lycaas@163.com (Y.L.); weidongzhu17@163.com (W.Z.); m13663726262@163.com (X.F.); wangjj@cricaas.com.cn (J.W.); linhuan0829@163.com (H.L.); 2 State Key Laboratory of Crop Biology, College of Agronomy, Shandong Agricultural University, Taian 271000, Shandong, China; genetics@sdau.edu.cn

**Keywords:** upland cotton, TCP genes, abiotic stress, miR319, target genes

## Abstract

The TEOSINTE BRANCHED 1, CYCLOIDEA, and PROLIFERATING CELL FACTORS (TCP) gene family is a group of plant-specific transcription factors that have versatile functions in developmental processes and stress responses. In this study, a total of 73 *TCP* genes in upland cotton were identified and characterizated. Phylogenetic analysis classified them into three subgroups: 50 belonged to PCF, 16 to CIN, and 7 to CYC/TB1. *GhTCP* genes are randomly distributed in 22 of the 26 chromosomes in cotton. Expression patterns of *GhTCPs* were analyzed in 10 tissues, including different developmental stages of ovule and fiber, as well as under heat, salt, and drought stresses. Transcriptome analysis showed that 44 *GhTCP* genes exhibited varied transcript accumulation patterns in the tested tissues and 41 *GhTCP* genes were differentially expressed in response to heat, salt, and drought stresses. Furthermore, three *GhTCP* genes of the CIN clade were found to contain miR319-binding sites. An anti-correlation expression of *GhTCP21* and *GhTCP54* was analyzed with miR319 under salt and drought stress. Our results lay the foundation for understanding the complex mechanisms of GhTCP-mediated developmental processes and abiotic stress-signaling transduction pathways in cotton.

## 1. Introduction

Transcription factors are essential for the control of gene expression. Gene expression can be regulated by transcription factors that either activate or repress transcription, so they are vital for many cell biological process [1]. The TEOSINTE BRANCHED 1, CYCLOIDEA, and PROLIFERATING CELL FACTORS (TCP) gene family is a small group of transcription factors exclusive to higher plants [2]. This class of transcription factors has many functions in regulating diverse plant growth and development processes by controlling cell proliferation [3]. They are characterized by a highly conserved 59-amino-acid basic helix–loop–helix (bHLH) motif at the N-terminus designated as the TCP domain [4]. This domain is responsible for DNA binding, nuclear targeting, and is involved in protein–protein interactions [5]. Based on variation in the TCP domain, TCP family members can be classified into two classes: Class I (also known as the PCF or TCP-P class) and class II (also known as the TCP-C class) [6,7]. Class II is further subdivided into the CINCINNATA (CIN) and CYC/TB1 subgroups. In addition to the TCP domain, several class II members possess an 18–20-residue arginine-rich motif [8]. This so-called R domain was predicted to form a hydrophilic α-helix or a coiled-coil structure that mediates protein–protein interactions [2].

It has been reported that many TCP transcription factors participate in the regulation of diverse physiological and biological processes, such as phytohormone biosynthesis and signal transduction, branching, leaf morphogenesis flower development and senescence, pollen development, and regulation of the circadian clock in various plants [9,10,11]. In *Arabidopsis thaliana* seeds, *TCP14* was expressed in the vascular tissues of embryos. It promotes germination through antagonism of abscisic acid signaling [12]. *TCP1* is expressed in restricted areas of the flower meristem, leaf vasculature, and at the junctions of roots and hypocotyls. It mediates the expression of a key brassinosteroid (BR) biosynthetic gene by directly associating with the two GGNCCC motifs in the promoter region of *DWARF4* (*DWF4*) [13]. *DWF4* encodes a 22-hydroxylase and is responsible for multiple 22-hydroxylation steps during BR biosynthesis [14]. The expression levels of *DWF4* were positively correlated with *TCP1* abundance in *planta*. In Arabidopsis flowers, the gynoecium and silique development was modulated by *TCP15* through partly regulating auxin biosynthesis. The ectopic expression of Arabidopsis *TCP15* represses style and stigma development, thus producing gynoecia with decreased stigmatic tissue and/or carpel fusion defects in apical parts [15]. TCP17 and its two closely related homologs, TCP5 and TCP13, play an important role in mediating shade-induced hypocotyl elongation by up-regulating auxin biosynthesis via a PHYTOCHROME INTERACTING FACTORS (PIF)-dependent and a PIF-independent pathway [16]. In rice (*Oryza sativa*), *OsTCP19* was upregulated under salt and water-deficit stress. Overexpression of *OsTCP19* in Arabidopsis caused upregulation of *INDOLE-3-ACETIC ACID3*(*IAA3*), *ABSCISIC ACID INSENSITIVE 3*(*ABI3*), and *ABI4,* and downregulation of *LIPOXYGENASE2* (*LOX2*), thus leading to developmental abnormalities, such as less lateral roots [17]. MicroRNAs (miRNAs) are a class of smallnon-coding RNAs generated from single-strand hairpin RNA precursors. They regulate gene expression by binding to complementary sequences within target mRNAs [18]. Considerable progress has been made in identifying the targets of plant miRNAs. In Arabidopsis, five CIN-like *TCP* genes (*TCP2*, *TCP3*, *TCP4*, *TCP10*, and *TCP24*) were targeted by miR319 and have been implicated in regulating leaf morphogenesis [19]. Knockdown of a subset of Class II TCP transcription factors by overexpression of miR319 increases tolerance to dehydration and salinity stress in bentgrass (*Agrostis stolonifera*) [20]. Accumulated functional characterization of TCPs indicated their diverse function in a developmental-, tissue-, and signal-dependent context. In addition to their importance as transcriptional regulators of cell-cycle genes, TCPs have other functions with comparable impact on plant development. Characterization of TCPs and their signaling pathway will be beneficial to unravel their exact role in the control of plant development and evolution.

Allotetraploid upland cotton (*Gossypiumhirsutum* L.) accounts for more than 90% of cultivated cotton worldwide, is the main source of renewable textile fibers, and is also grown to produce oilseed. It has proven to be difficult to sequence, owing to its complex allotetraploid (A_t_D_t_) genome [21]. Recently, its whole genome was sequenced by integrating whole-genome shotgun reads, bacterial artificial chromosome-end sequences, and genotype-by-sequencing genetic maps [22,23]. Repeated sequences account for 67.2% of the A_t_D_t_ genome, and transposable elements originating from D_t_ were more active than those from A_t_ [23]. Availability of the genome information can provide a great opportunity to identify and characterize TCP genes in this plant species for the first time. In this study, we identified and characterized 73 non-redundant TCP transcription factors in the *G. hirsutum* genome. Detailed information regarding their genomic structures, chromosomal locations, and a phylogenetic tree were also provided. Using RNA-seq data, we investigated their transcript profiles in different tissues, including different developmental stages of ovule and fiber, as well as their response to heat, drought, and salt stress. Furthermore, the miR319-targeted TCP genes were characterized.

## 2. Results

### 2.1. Identification and Characterization of TCP Proteins in G. hirsutum

To identify the *TCP* genes in the *G. hirsutum* genome, protein sequences of Arabidopsis and rice TCPs serve as BLAST search queries, and multiple-alignment was performed. A total of 73 *TCP* genes were identified. All candidate TCP genes were confirmed to encode the conserved TCP domain using the InterProScan database and NCBI’s CDD, the Conserved Domain Database [24]. Seven *GhTCP* genes were found to possess the R domain. These characteristic features suggested that they were members of the TCP gene family. Detailed characteristics of the TCP transcription factors in *G. hirsutum* are offered in Table S1. The GhTCP proteins are different in their length, molecular weight (Mw), and theoretical isoelectric point (pI). The mean length and Mw of these proteins was 347 amino acids and 37.58 kDa, respectively. The pI varied from pH 5.80 (GhTCP9) to 10.07 (GhTCP38) with an average of pH 8.09. All the GhTCP proteins were predicted to localize in the nucleus. Proteins were localized at their appropriate subcellular compartment to perform their desired function [3,25].

**Figure 1:**
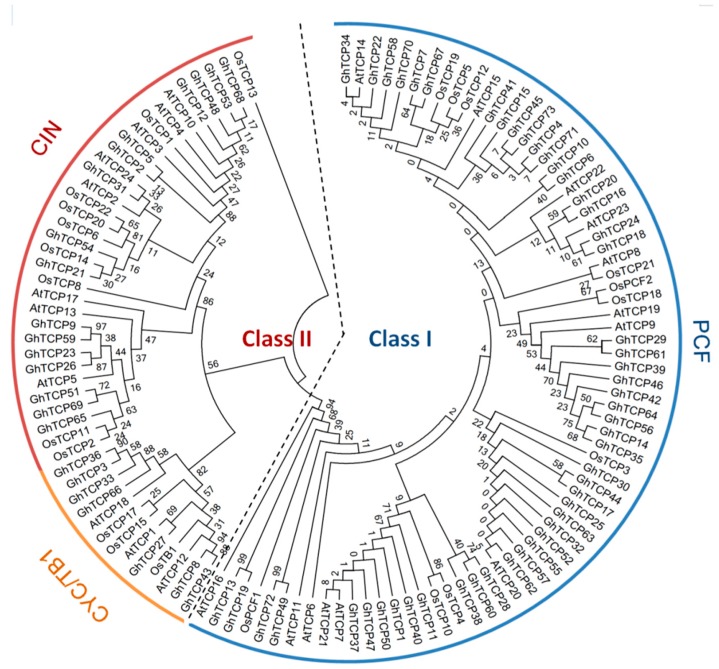
Phylogenetic analysis of TCP proteins from *G. hirsutum*, *Arabidopsis*, and rice. The deduced full-length amino acid sequences were aligned using ClustalX 2.0 and the phylogenetic tree was constructed using MEGA 6.0 by the Neighbor-Joining (NJ) method with 1000 bootstrap replicates. The three subclasses are indicated with different colors.

**Figure 2:**
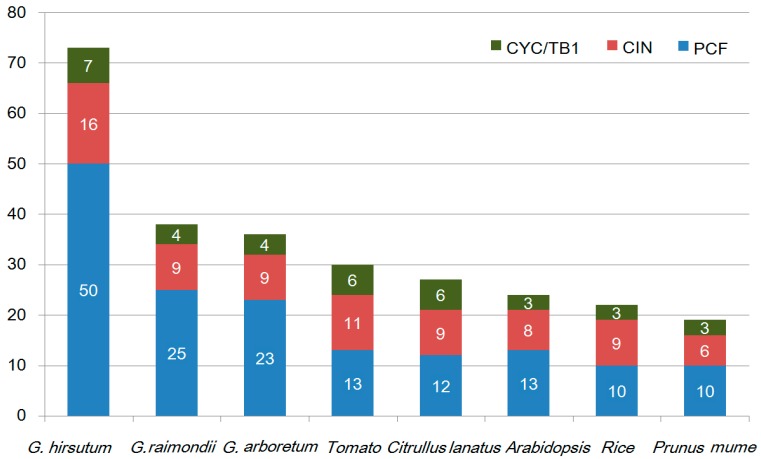
TCP family members of *G. hirsutum*, *G. raimondii*, *G. arboretum*, tomato, *Citrullus lanatus*, Arabidopsis, rice, and *Prunus mume*. Different colors represent the different subclasses, and the number of genes in each subclass is shown. Green: *CYC/TB1* genes; Red: *CIN* genes; Blue: *PCF* genes.

Unrooted phylogenetic trees were constructed based on the multiple sequence alignment of 73 GhTCP protein sequences and their Arabidopsis and rice homologs. The TCP transcription factors from the three species were distributed in almost all clades, indicating that the TCP family diversified before divergence of these plants. The phylogenetic tree placed the GhTCPs into two classes (Figure 1), as was also found for all species so far. Class I was named the TCP-P or PCF class, and class II was named the TCP-C class. The class II genes were further divided into two groups: CYC/TB1 and CIN. In *G. hirsutum*, CYC/TB1 and CIN were a larger family: For CYC/TB1, approximately twice the size of those of Arabidopsis and rice; and for CIN, approximately five times the size. Seven GhTCP genes belonged to the CYC/TB1 group–in Arabidopsis and rice, three of 24 AtTCPs and three of 21 OsTCPs were grouped into this subfamily. Fifty GhTCPs belonged to the PCF group, and13 AtTCPs and10 OsTCPs were also grouped into this subfamily. CYC/TB-type proteins were divided into two subgroups. One group contained four *G. hirsutum* TCPs, but only one Arabidopsis TCP and none from rice, which indicated that this group was either acquired after the divergence of monocots and dicots or was lost in rice. In *G. hirsutum*, the number of TCP genes was significantly higher than those in tomato, *Citrullus lanatus*, *Arabidopsis*, rice, and *Prunus mume* (Figure 2).

### 2.2. Genomic Distribution, Gene Structural Organization, and Domain Analysis of GhTCP Genes

The complete genome sequences provided an overview of the chromosomal distribution of these TCP genes. Among the 73 *G. hirsutum* TCPs, 67 members were located on the 22 chromosomes, and the other six were located at six unmapped scaffolds. *GhTCP* genes were unevenly distributed on 22 of the 26 *G. hirsutum* chromosomes, with the number of *TCP* genes per chromosome in the range of 0–8 (Figure S1). Chromosomes, A12 and D11, contained eight and seven genes, respectively, while chromosomes A02, A06, D03, D06, and D13 had no TCP genes.

To better understand the gene structures of GhTCP family genes, we analyzed their exon–intron organization. Overall, 88% of the GhTCPs contained only one exon (Figure S2). Seven GhTCP genes contained one intron and two exons: *GhTCP3*, *GhTCP23*, *GhTCP26*, *GhTCP56*, *GhTCP64*, and *GhTCP66*. Only *GhTCP33* in the CYC/TB1 group possessed four introns and five exons. Losses or gains of exons were identified during the evolution of the PCF group genes. *GhTCP19* comprised seven introns and eight exons, whereas *GhTCP13* consisted of four introns and five exons. Comparing their structural patterns showed the loss of an exon in the middle of the *GhTCP13* sequence. Two PCF class genes contained one intron and two exons, and the remaining PCF class genes contained only one exon. Analysis of the pattern of exon–intron junctions can provide important understanding into the evolution of gene families. Our results suggested that TCP genes maintained a relatively constant exon–intron composition during evolution of the *G. hirsutum* genome.

The conserved motif of TCP proteins in *G. hirsutum* was investigated using Clustal X. The sequences were found to encode a putative TCP-domain protein that contained a bHLH-type motif at the N-terminus (Figure S3). The components of the loop, and helixes I and II, were quite different between class I and II proteins. Within the TCP domain, several putative residues involved in DNA binding were located in the basic region and several putative hydrophobic residues located in helixes I and II. In the basic region, the CIN and CYC/TB1 type proteins contained an insertion of four amino acids. The R domain, an arginine-rich motif of 18–20 residues, was absent from all class I proteins and was mainly present in CYC/TB1 group proteins.

### 2.3. Expression Analysis of GhTCP Genes in Different Tissues and under Various Stress Conditions

**Figure 3:**
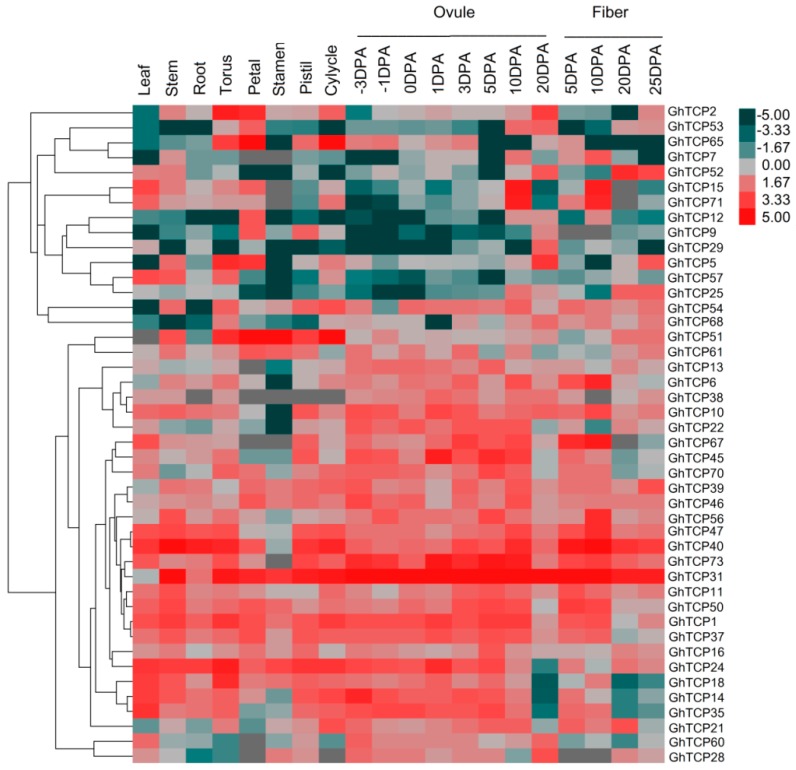
Heat map representation of GhTCP gene expression in different tissues. The tissues used for expression profiling are indicated at the top. The genes are shown on the left of the expression bars and the phylogenetic relationship is shown. The −3 to 20 days post anthesis (DPA) indicate −3, −1, 0, 1, 3, 5, 10, and 20 days after pollination.

To provide reliable information on the growth and developmental functions of TCP genes in *G. hirsutum*, their transcript accumulation patterns in mature leaves, stem, root, torus, petal, stamen, pistil, cylycle, ovules, and fibers of *G. hirsutum* was investigated (Figure 3). We obtained transcriptome data from the NCBI Sequence Read Archive (accession number RJNA248163). Some TCP genes with close phylogenetic relationships showed similar or divergent expression patterns. For instance, the paralogous pair, *GhTCP2* and *GhTCP5*, was expressed highly in both the torus and petal, at moderate levels in the ovules at 20 days post anthesis (DPA), and at low levels in mature leaves. Most CYC/TB1-type genes were only weakly or not expressed in all tissues, suggesting that they were primarily expressed in other organs not tested or under special conditions. In contrast, *GhTCP31*, *GhTCP40*, and *GhTCP47* were constitutively expressed at very high levels in all tissues tested, indicating that these genes played regulatory roles during multiple development stages. Some TCP genes exhibited tissue-specific expression. *GhTCP31* and *GhTCP40* were highly expressed only in reproductive organs: Torus, petal, stamen, pistil, and calycles.

**Figure 4:**
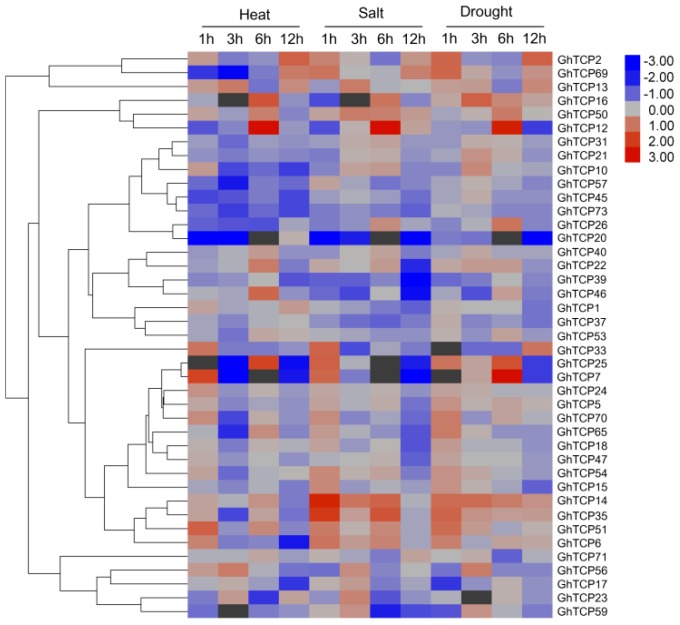
Expression of GhTCP genes under heat, salt, and drought stresses. The genes are shown on the left of the expression bars and the phylogenetic relationship is shown. The abiotic stresses used for expression profiling are indicated at the top. The 1, 3, 6, and 12 h indicate hours after treatment.

**Figure 5:**
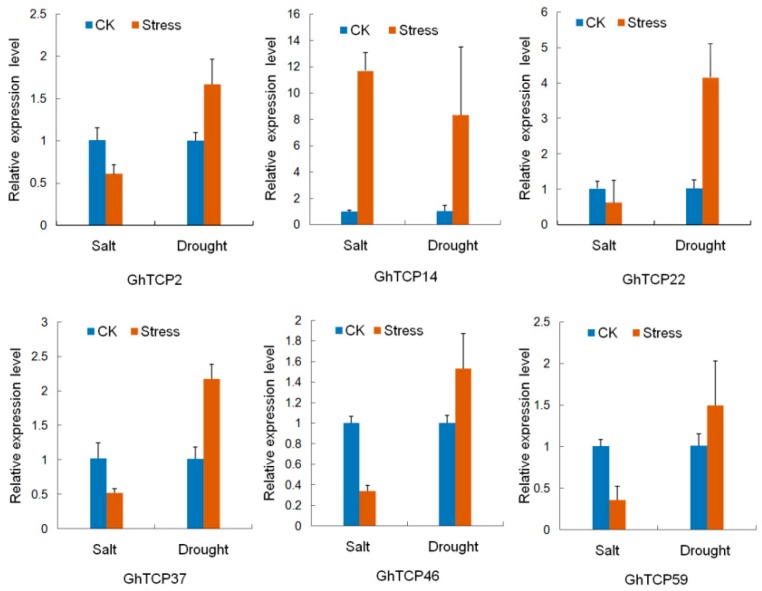
Relative expression levels of six *GhTCP* genes under salt and drought stress. QRT-PCR analyses were performed using RNA generated from cotton leaves after NaCl and Polyethylene Glycol (PEG) treatment. Error bars represent standard error of the mean.

To predict possible functions of *TCP* genes in environmental adaptation, we investigated the transcriptional profile of *TCP* genes under various stress conditions, including heat, salt, and drought stresses. In total, 41 genes exhibited variations in expression (Figure 4). Of the three treatments, heat stress caused relatively more fluctuations in the transcript abundance of *TCPs* than did salt or drought stress. Under heat stress conditions, 18 *TCP* genes were downregulated and eight were upregulated. In response to salt treatment, the expression of five GhTCPs (*TCP7*, *14*, *25*, *33*, and *35*) increased instantly, and then decreased slowly during continued salt stress. Six GhTCP genes were selected at random for quantitative RT-PCR (qRT-PCR) analysis to determine the relative expression under salt and drought stresses (Figure 5). The qRT-PCR results indicated that these *GhTCP* genes showed similar expression patterns to the transcriptome sequencing results.

### 2.4. Target Sites of miR319 in GhTCP Genes

**Figure 6:**
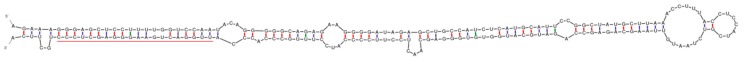
Mature and predicted fold-back structures of miR319 precursors in *G. hirsutum*. Sequences of mature miR319 are underlined.

The miRNAs can cause endonucleolytic cleavage of mRNA by extension, which often perfect complementarity to mRNAs. In plants, miR319 was one of the first characterized and conserved miRNA families, which has been demonstrated to target *TCP* genes. In *G. hirsutum*, miR319 had only 1-nt mismatch compared with sequences in Arabidopsis (Figure 6). The predicted hairpin structures of the miR319 precursor had 191 nt. The miR319 sequence was located at the 3′-end of the pre-miRNAs and began with a 5′-uridine. Using a set of strict standards, *GhTCP21*, *GhTCP31*, and *GhTCP54* were predicted as targets of miR319. These three miR319 target sites were all located in the coding regions, and all miR319-targeted genes belonged to the CIN clade. Similarly, there were five and three *TCP* genes containing miR319-binding sites in Arabidopsis and *P. mume*, respectively, and they also belonged to the CIN group [26]. This suggests that miR319 held homologous target interactions during the evolution and diversification of plants.

**Figure 7:**
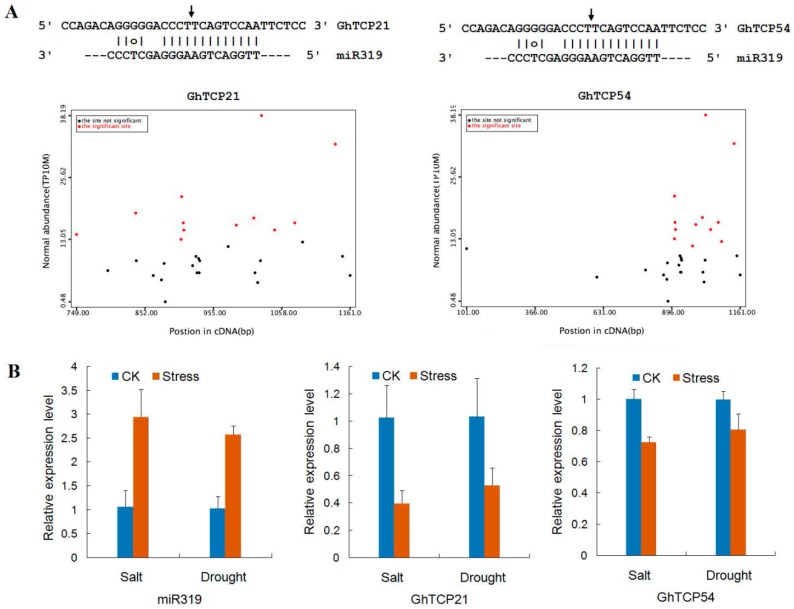
miR319 and its target genes, *GhTCP21* and *GhTCP54,* in *G. hirsutum*. (**A**) Target plot (t-plot) for *GhTCP21* and *GhTCP54*, which were targeted by miR319. Arrows indicate the signatures corresponding to the miRNA cleavage site. Partial mRNA sequences of target genes aligned with the miRNAs show perfect matches (straight lines) and G-U wobbles (circles). (**B**) Relative expression levels of miR319, *GhTCP21*, and *GhTCP54* under salt and drought stresses. QRT-PCR analyses were performed using RNA generated from cotton leaves after NaCl and Polyethylene Glycol (PEG) treatment. Error bars represent standard error of the mean.

Degradome sequencing had been widely used to identify plant miRNA cleavage sites. In this study, the *GhTCP* mRNA degradation sites were determined by BLASTing the sequenced degraded fragments against the *G. hirsutum* TCP genes. The degradome sequencing data are available at Gene Expression Omnibus (GEO accession number GSE69820). Using PairFinder software, miR319 was found to cleave *GhTCP21* and *GhTCP54* mRNA transcripts (Figure 7). The miR319–mRNA pair was at the cleavage site of the two TCP genes. There were both 67 raw reads at the position, with abundance at the position equal to the maximum on the transcript, and with only one maximum on the transcript. The 5′-ends of the mRNA fragments mapped to the nucleotide that paired to the tenth nucleotide of the miR319 sequence. To research the biological function of miR319, a negative-correlation expression test was undertaken for miR319 and its target *GhTCP21* and *GhTCP54* mRNAs using qRT-PCR. In response to salt and drought treatments, miR319 showed different degrees of upregulation. The transcriptome sequencing analysis showed that expression of *GhTCP21* and *GhTCP54* was downregulated.

## 3. Discussion

A number of TCP proteins had been recently identified in various plants due to completion of their whole-genome sequence, including *Arabidopsis*, rice, tomato (*Solanum lycopersicum*), and watermelon (*Citrullus lanatus*), *Orchis italica*, and *Populus euphratica* [27,28,29,30,31]. The allotetraploid, *G. hirsutum*, is not only the world’s most important fiber crop, but is also a model polyploid crop. Despite being among the largest and most diverse gene families, the TCP gene family has not been systematically identified in the *G. hirsutum* genome. In this study, we identified 73 *TCP* genes in the sequenced genome of *G. hirsutum*. We analyzed their phylogenetic relationship, genomic distribution, conserved protein motif, and exon–intron organization. Over 80% of *GhTCP* genes were intronless, which was quite similar to the structure of *G. raimondii* and *G. arboretum TCP* genes [32,33]. Generally speaking, most *GhTCPs* within the same subclade showed similar gene structure in terms of numbers and lengths of introns and exons. Furthermore, similar to the exon–intron organization, members of the same subclade also showed similar motif composition, indicating their functional similarities. Additionally, some motifs were only present at specific subclades, such as the R domain, suggesting that they can have subclade-specific functions.

The *GhTCP* genes possessed an expanded family, with approximately three-fold size compared with *Arabidopsis*, tomato, and rice, and approximately two-fold compared with *G. arboreum* and *G. raimondii*. This suggests that although plant *TCP* genes may derive from a common ancestor, many had undergone distinct patterns of differentiation with the divergence of different lineages. Based mainly on amino acid sequence differences, especially in the basic region of the TCP domain, the TCP transcription factors are divided into three groups. There were 50 *GhTCP* genes in the PCF group, 16 in the CIN group, and seven in the CYC/TB1 group. The numbers of genes in each group were approximately twice those in *G. arboretum* and *G. raimondii*. According to a recent study, all tetraploid cotton species (A_t_D_t_) evolved from A-genome diploid, *G. arboretum,* and D-genome diploid, *G. raimondii*, at around 1–2 Mya [34]. In addition, previous studies indicated that gene duplication contributed to increasing the number of gene family members on various scales, including whole-genome duplication [35]. The expansion of regulatory genes is rarely achieved simply through single gene duplication alone, implying that genome duplication contributed to the amplification of the TCP gene family in *G. hirsutum*.

TCP transcription factors was involved in the regulation of cell growth and proliferation, which performed diverse functions in multiple aspects of plant growth and development [3]. We determined the spatial and temporal expression profiles of *G. hirsutum* TCP genes in 10 tissues, which included different developmental stages of ovule and fiber, using transcriptome analysis. The expression in different tissues varied widely among *GhTCP* genes and different organs for individual TCP genes. This implies functional divergence of *GhTCP* genes during different plant developmental processes. *GhTCP15* and *GhTCP71* were relatively highly expressed in ovules and fibers at 10 DPA. Previous study demonstrated that *GbTCP* was preferentially expressed in elongating *G. barbadense* fiber during5 to 15 DPA [36]. Overexpression of *GbTCP* enhanced root hair initiation and elongation in *Arabidopsis* and regulated branching. Both *GbTCP* in *G. barbadense* and *GhTCP71* are orthologs of *AT1G69690* in *Arabidopsis* (named *AtTCP15*), compared with which they had only one amino acid difference within the TCP domain [37]. *AtTCP15* was expressed in trichomes and rapidly dividing tissues and vascular tissue, and the protein promoted mitotic cell division, but inhibited endo-reduplication by modulating the expression of several key cell-cycle genes [15,38]. In our study, *GhTCP14* was also expressed predominantly in fiber cells, especially at the initiation and elongation stages of development as previously reported. Induced expression of *GhTCP14* can increases the density and length of root hairs and trichomes and affects gravitropism of *Arabidopsis* [39]. These results suggested that cotton fiber and *Arabidopsis* root hair elongation may have a similar regulatory mechanism for *TCP* genes.

Many *Arabidopsis* TCP genes with similar functions tended to cluster in the same clade, implying that TCP genes within the same clade may have similar functions in *G. hirsutum*. In *Arabidopsis*, some angiosperm members of the CIN-like clade involved in leaf and flower morphogenesis are targeted by miR319–for example, *AtTCP2*, *3*, *4*, *10*, and *24* [40]. Loss of function of these genes results in enlarged leaves, due to an excess of cells that are smaller in size, while their gain of function leads to smaller leaves [41,42]. The miR319, previously known as “miR-JAW”, was first described in *Arabidopsis* because its involvement in the control of leaf morphogenesis [43]. Several studies had reported the involvement of miR319 in plants in response to stress conditions via downregulation of its target genes [44]. Transgenic creeping bentgrass overexpressing a rice miR319, *Osa-miR319a*, exhibited enhanced salt and drought tolerance [45]. In this study, we observed upregulation of miR319 and downregulation of the targets in both salt and drought treatments. To understand the responses of *GhTCP* genes to stresses, the expression profiles were investigated in response to abiotic stresses, such as heat, salinity, and drought. In total, 40 *GhTCP* genes exhibited variations in expression. It is noteworthy that some genes showed instantaneous upregulation, and decreased slowly during continued stress. For example, *GhTCP6*, *14*, *35*, and *51* exhibited their highest expression at 3 h of dehydration and salinity treatment. However, no significantly upregulated expression was found at late time points. It is plausible to postulate that these genes might be the part of a stress-signaling system. The functions of these stress-responsive *GhTCP* genes in abiotic stress resistance will be further characterized in future work.

In this study, a total of 73 non-redundant TCP encoding genes were identified in *G. hirsutum*. Our results provided evidence for the relationship between structure and function in the *G. hirsutum* TCP gene family, and laid the foundation for further identification of the functions of the *GhTCP* gene family and their relationship with miR319.

## 4. Materials and Methods

### 4.1. Plant Materials and Treatments

The *G. hirsutum* L. accession TM-1 was used in this study. The seeds were provided by the National Mid-term Genebank of the Institute of Cotton Research in China. Cotton seeds were sterilized, and germinated in vermiculite under greenhouse conditions: 30/22 °C day/night temperature, 55–70% relative humidity, and a 14/10 h light/dark cycle under 450 μmol m^−2^·s^−1^ light intensity. At the two-leaf stage, healthy seedlings were placed in pots containing aerated nutrient solution. Plants were cultured under normal conditions for 10 d to ensure full establishment before starting the drought and salt stress treatments. The pH was maintained close to 6.9 by adding H_2_SO_4_ or KOH as required. The roots of cotton seedlings were irrigated with 20% PEG to test the response to drought. The seedlings were treated with 150 Mm NaCl solution to test the response to salt. After exposing the seedlings to drought and salt stress for 24 h, leaves were harvested directly into liquid nitrogen and stored at −80 °C for subsequent use.

### 4.2. Sequence Retrieval and TCP Gene Identification

To identify TCPs in *G. hirsutum*, multiple database searches were performed. The completed genome sequence and protein sequences of this species were downloaded from the CottonGen database (http://www.cottongen.org) and the Cotton Genome Project (http://cgp.genomics.org.cn/page/species/index.jsp). A local protein database was constructed using the protein sequences. The TCP proteins from *Arabidopsis* and rice were used as query sequences, and were collected from published literature and downloaded from The *Arabidopsis* Information Resource (TAIR release 10, http://www.arabidopsis.org) and the Rice Genome Annotation Project (ftp://ftp.plantbiology.msu.edu), respectively. The BLASTP (http://cgp.genomics.org.cn/) was used to do the BLAST search. The e-value was set at 1e-10. The candidate *TCP* genes were further aligned to remove redundant sequences. To verify the reliability of the initial results, all non-redundant candidate TCP sequences were analyzed to confirm the presence of the conserved TCP domain using the InterProScan database (https://www.ebi.ac.uk/) and the NCBI’s CDD (http://www.ncbi.nlm.nih.gov/Structure/cdd/cdd.shtml). Based on the results, the sequences that did not include the TCP domain were eliminated.

### 4.3. Analysis of Protein Features and Chromosomal Locations

The Mw and pI of each TCP protein were obtained using the online ExPASy program (http://web.expasy.org/compute_pi/). Protein pI was calculated using pK values of amino acids. Protein Mw was calculated by the addition of average isotopic masses of amino acids in the protein and the average isotopic mass of one water molecule. The subcellular localization of each GhTCP protein was analyzed using the CELLO v2.5 server (http://cello.life.nctu.edu.tw/). Through BLASTN (http://cgp.genomics.org.cn/) searches against the *G. hirsutum* whole genome, some information was obtained about the physical locations of each *GhTCP* genes on chromosomes.

### 4.4. Phylogenetic Analysis and Gene Structure

To analyzethe phylogenetic relationships between *TCP* genes in *G. hirsutum* and other species, the protein sequences of the identified *GhTCP* genes, Arabidopsis *TCP* genes, and rice *TCP* genes, were used to generate aphylogenetic tree. The ClustalX program was used to align the TCP domains. Phylogenetic trees were constructed by MEGA6.0 using the NJ and Minimal Evolution (ME) methods. For both methods, the bootstrap test of phylogeny was performed with 1000 replications. The exon/intron structures for each *GhTCP* gene was determined by aligning the CDS sequences to their corresponding genomic DNA sequences. The structures were shown using the Gene Structure Display Server 2.0 (http://gsds.cbi.pku.edu.cn/).

### 4.5. Expression Analyses of the TCP Genes and Search for miR319 Targets

Expression data for *GhTCP* genes were obtained from transcriptome data. RNA-seq data were obtained from the NCBI Sequence Read Archive (SRA: PRJNA248163). The expression pattern of *GhTCP* genes was analyzed in leaves, roots, and stems of 2-week-old plants; petals, torus, pistils, stamens, and lower sepals dissected from whole mature flowers; ovules from −3, −1, 0, 1, 3, 5, 10, and 20 days after pollination; fibers from 5, 10, 20, and 25 days; and true leaves of seedlings treated with salt, PEG, and heat. Gene expression levels were calculated according to Fragments Per Kilobase Million (FPKM) values and the default empirical abundance threshold of FPKM > 1 was used to identify the expressed gene.

Degradome sequencing data were used to find miR319 that caused TCP transcript degradation (GEO: GSE69820). We matched the degraded fragments to the GhTCP gene sequences, identified the cDNA sequences expressed, and then calculated normalized expression numbers of each degraded site along every cDNA, blast with miR319 sequences. A t-plot figure was constructed to show the tag distributions. PairFinder software was used to identify the sliced targets for miRNAs.

### 4.6. RNA Extraction and qRT-PCR Analysis

Total RNA was extracted with TRIzol Reagent (Invitrogen, 15596-026, Dalian, China) according to the manufacturer’s instructions. For the first-strand cDNA synthesis experiment of miR319, a One Step PrimeScript^®^ miRNA cDNASynthesis Kit (Takara, Dalian, China) was used. For each sample, 4 μg of total RNA was converted to cDNA in a 20-μL reaction system, which contained 10 μL of 2× miRNA reaction buffer mix, 2 μL of 0.1% BSA, and 2 μL of miRNA PrimeScript^®^RT Enzyme Mix. qRT-PCR was performed using SYBR^®^ Premix Ex TaqTM II (Takara) and undertaken with a 7500 Fast Real-Time PCR system (Applied Biosystems Inc., Foster City, CA, USA). The specific miR319 and TCP genes primers used are given in Table S2. The reactions were incubated in a 96-well plate at 95 °C at 30 s, followed by 40 cycles of 95 °C at 15 s and 60 °C at 30 s. The 25-μL reaction solutions contained 12.5 μL of SYBR^®^*Premix* Ex Taq^TM^II (2×), 1 μL of PCR forward primer (10 μM), 1 μL of PCR reverse primer (10 μM) and 2 μL of five fold diluted cDNA template. All reactions were performed with three replicates. Relative expression levels were calculated by the comparative threshold cycle (2^−ΔΔT^) method.

## Supplementary Materials

Supplementary materials can be found at http://www.mdpi.com/1422-0067/19/11/3655/s1.

## References

[1] Lee T.I., Young R.A. (2000). Transcription of eukaryotic protein-coding genes. Annu. Rev. Genet..

[2] Cubas P., Lauter N., Doebley J., Coen E. (1999). The TCP domain: A motif found in proteins regulating plant growth and development. Plant J..

[3] Martin-Trillo M., Cubas P. (2010). TCP genes: A family snapshot ten years later. Trends Plant Sci..

[4] Aggarwal P., Das Gupta M., Joseph A.P., Chatterjee N., Srinivasan N., Nath U. (2010). Identification of specific DNA binding residues in the TCP family of transcription factors in Arabidopsis. Plant Cell.

[5] Kosugi S., Ohashi Y. (2002). DNA binding and dimerization specificity and potential targets for the TCP protein family. Plant J..

[6] Navaud O., Dabos P., Carnus E., Tremousaygue D., Herve C. (2007). TCP transcription factors predate the emergence of land plants. J. Mol. Evol..

[7] Viola I.L., Reinheimer R., Ripoll R., Manassero N.G., Gonzalez D.H. (2012). Determinants of the DNA binding specificity of class I and class II TCP transcription factors. J. Biol. Chem..

[8] Sarvepalli K., Nath U. (2018). CIN-TCP transcription factors: Transiting cell proliferation in plants. IUBMB Life.

[9] Kosugi S., Ohashi Y. (1997). PCF1 and PCF2 specifically bind to cis elements in the rice proliferating cell nuclear antigen gene. Plant Cell.

[10] Li C., Potuschak T., Colon-Carmona A., Gutierrez R.A., Doerner P. (2005). Arabidopsis TCP20 links regulation of growth and cell division control pathways. Proc. Nat. Acad. Sci. USA.

[11] Danisman S. (2016). TCP Transcription Factors at the Interface between Environmental Challenges and the Plant’s Growth Responses. Front. Plant Sci..

[12] Tatematsu K., Nakabayashi K., Kamiya Y., Nambara E. (2008). Transcription factor AtTCP14 regulates embryonic growth potential during seed germination in Arabidopsis thaliana. Plant J..

[13] Gao Y., Zhang D., Li J. (2015). TCP1 Modulates DWF4 Expression via Directly Interacting with the GGNCCC Motifs in the Promoter Region of DWF4 in Arabidopsis thaliana. J. Genet. Genom..

[14] Kim H.B., Kwon M., Ryu H., Fujioka S., Takatsuto S., Yoshida S., An C.S., Lee I., Hwang I., Choe S. (2006). The regulation of DWARF4 expression is likely a critical mechanism in maintaining the homeostasis of bioactive brassinosteroids in Arabidopsis. Plant Physiol..

[15] Lucero L.E., Uberti-Manassero N.G., Arce A.L., Colombatti F., Alemano S.G., Gonzalez D.H. (2015). TCP15 modulates cytokinin and auxin responses during gynoecium development in Arabidopsis. Plant J..

[16] Zhou Y., Zhang D.Z., An J.X., Yin H.J., Fang S., Chu J.F., Zhao Y.D., Li J. (2018). TCP Transcription Factors Regulate Shade Avoidance via Directly Mediating the Expression of Both PHYTOCHROME INTERACTING FACTORs and Auxin Biosynthetic Genes. Plant Physiol..

[17] Mukhopadhyay P., Tyagi A.K. (2015). OsTCP19 influences developmental and abiotic stress signaling by modulating ABI4-mediated pathways. Sci. Rep..

[18] Carrington J.C., Ambros V. (2003). Role of microRNAs in plant and animal development. Science.

[19] Schommer C., Palatnik J.F., Aggarwal P., Chetelat A., Cubas P., Farmer E.E., Nath U., Weigel D. (2008). Control of jasmonate biosynthesis and senescence by miR319 targets. PLoS Biol..

[20] Zhou M., Li D., Li Z., Hu Q., Yang C., Zhu L., Luo H. (2013). Constitutive expression of a miR319 gene alters plant development and enhances salt and drought tolerance in transgenic creeping bentgrass. Plant Physiol..

[21] Chen Z.J., Scheffler B.E., Dennis E., Triplett B.A., Zhang T., Guo W., Chen X., Stelly D.M., Rabinowicz P.D., Town C.D. (2007). Toward sequencing cotton (Gossypium) genomes. Plant Physiol..

[22] Zhang T., Hu Y., Jiang W., Fang L., Guan X., Chen J., Zhang J., Saski C.A., Scheffler B.E., Stelly D.M. (2015). Sequencing of allotetraploid cotton (Gossypium hirsutum L. acc. TM-1) provides a resource for fiber improvement. Nat. Biotechnol..

[23] Li F., Fan G., Lu C., Xiao G., Zou C., Kohel R.J., Ma Z., Shang H., Ma X., Wu J. (2015). Genome sequence of cultivated Upland cotton (Gossypium hirsutum TM-1) provides insights into genome evolution. Nat. Biotechnol..

[24] Marchler-Bauer A., Zheng C., Chitsaz F., Derbyshire M.K., Geer L.Y., Geer R.C., Gonzales N.R., Gwadz M., Hurwitz D.I., Lanczycki C.J. (2013). CDD: Conserved domains and protein three-dimensional structure. Nucleic Acids Res..

[25] Qin L.J., Guo X.Z., Feng X.Z., Weng L., Yan J., Hu X.H., Luo D. (2004). Cloning of LjCYC1 gene and nuclear localization of LjCYC1 protein in Lotus japonicus. Zhi Wu Sheng Li Yu Fen Zi Sheng Wu Xue Xue Bao.

[26] Zhou Y., Xu Z., Zhao K., Yang W., Cheng T., Wang J., Zhang Q. (2016). Genome-Wide Identification, Characterization and Expression Analysis of the TCP Gene Family in Prunus mume. Front. Plant Sci..

[27] Parapunova V., Busscher M., Busscher-Lange J., Lammers M., Karlova R., Bovy A.G., Angenent G.C., de Maagd R.A. (2014). Identification, cloning and characterization of the tomato TCP transcription factor family. BMC Plant Biol..

[28] De Paolo S., Gaudio L., Aceto S. (2015). Analysis of the TCP genes expressed in the inflorescence of the orchid Orchis italica. Sci. Rep..

[29] Ma X., Ma J., Fan D., Li C., Jiang Y., Luo K. (2016). Genome-wide Identification of TCP Family Transcription Factors from Populus euphratica and Their Involvement in Leaf Shape Regulation. Sci. Rep..

[30] Shi P., Guy K.M., Wu W., Fang B., Yang J., Zhang M., Hu Z. (2016). Genome-wide identification and expression analysis of the ClTCP transcription factors in Citrullus lanatus. BMC Plant Biol..

[31] Yao X., Ma H., Wang J., Zhang D. (2007). Genome-Wide Comparative Analysis and Expression Pattern of TCP Gene Families in Arabidopsis thaliana and Oryza sativa. J. Integrat. Plant Biol..

[32] Ma J., Wang Q., Sun R., Xie F., Jones D.C., Zhang B. (2014). Genome-wide identification and expression analysis of TCP transcription factors in Gossypium raimondii. Sci. Rep..

[33] Ma J., Liu F., Wang Q., Wang K., Jones D.C., Zhang B. (2016). Comprehensive analysis of TCP transcription factors and their expression during cotton (Gossypium arboreum) fiber early development. Sci. Rep..

[34] Paterson A.H., Wendel J.F., Gundlach H., Guo H., Jenkins J., Jin D., Llewellyn D., Showmaker K.C., Shu S., Udall J. (2012). Repeated polyploidization of Gossypium genomes and the evolution of spinnable cotton fibres. Nature.

[35] Kurosaki M., Bolis M., Fratelli M., Barzago M.M., Pattini L., Perretta G., Terao M., Garattini E. (2013). Structure and evolution of vertebrate aldehyde oxidases: From gene duplication to gene suppression. Cell Mol. Life Sci..

[36] Hao J., Tu L., Hu H., Tan J., Deng F., Tang W., Nie Y., Zhang X. (2012). GbTCP, a cotton TCP transcription factor, confers fibre elongation and root hair development by a complex regulating system. J. Exp. Bot..

[37] Kieffer M., Master V., Waites R., Davies B. (2011). TCP14 and TCP15 affect internode length and leaf shape in Arabidopsis. Plant J..

[38] Li Z.Y., Li B., Dong A.W. (2012). The Arabidopsis transcription factor AtTCP15 regulates endoreduplication by modulating expression of key cell-cycle genes. Mol. Plant.

[39] Wang M.Y., Zhao P.M., Cheng H.Q., Han L.B., Wu X.M., Gao P., Wang H.Y., Yang C.L., Zhong N.Q., Zuo J.R. (2016). The cotton transcription factor TCP14 functions in auxin-mediated epidermal cell differentiation and elongation. Plant Physiol..

[40] Mendez-Vigo B., de Andres M.T., Ramiro M., Martinez-Zapater J.M., Alonso-Blanco C. (2010). Temporal analysis of natural variation for the rate of leaf production and its relationship with flowering initiation in Arabidopsis thaliana. J. Exp. Bot..

[41] Koyama T., Furutani M., Tasaka M., Ohme-Takagi M. (2007). TCP transcription factors control the morphology of shoot lateral organs via negative regulation of the expression of boundary-specific genes in Arabidopsis. Plant Cell.

[42] Efroni I., Blum E., Goldshmidt A., Eshed Y. (2008). A protracted and dynamic maturation schedule underlies Arabidopsis leaf development. Plant Cell.

[43] Palatnik J.F., Allen E., Wu X., Schommer C., Schwab R., Carrington J.C., Weigel D. (2003). Control of leaf morphogenesis by microRNAs. Nature.

[44] Thiebaut F., Rojas C.A., Almeida K.L., Grativol C., Domiciano G.C., Lamb C.R., de Engler J.A., Hemerly A.S., Ferreira P.C. (2012). Regulation of miR319 during cold stress in sugarcane. Plant Cell Environ..

[45] Zhou M., Luo H. (2014). Role of microRNA319 in creeping bentgrass salinity and drought stress response. Plant Signal. Behav..

