# Supplementary material for: Identification, Characterization, and Expression Patterns of TCP Genes and microRNA319 in Cotton
Source: Int J Mol Sci. 2018 Nov 20;19(11):3655. doi: 10.3390/ijms19113655 (PMC6274894; doi:10.3390/ijms19113655)
Supplement: Supplementary file 1 [file ijms-19-03655-s001.zip › ijms-381771-supplementary/ijms-381771-supplementary.docx]

**Supplementary Materials:**

**Figure S1.**Locations of TCP genes on *G. hirsutum* chromosomes.

**Figure S2.** Structural analysis of *G. hirsutum* TCP transcription factors. Introns and exons are represented by black lines and colored boxes, respectively. TCP genes are grouped according to phylogenetic classification.

**Figure S3.**Multiple sequence alignment of *G. hirsutum* TCP transcription factors. Alignment was performed using the ClustalX program.

**Table S1**. Characteristics of TCP genes from cotton. AA: Amino acid; pI: The theoretical isoelectric point of proteins; Mw: The theoretical molecular weight of proteins.

**Table S2.** Primers used in real-time quantitative RT-PCR.
