# Supplementary material for: Identification, Characterization, and Expression Patterns of TCP Genes and microRNA319 in Cotton
Source: Int J Mol Sci. 2018 Nov 20;19(11):3655. doi: 10.3390/ijms19113655 (PMC6274894; doi:10.3390/ijms19113655)
Supplement: Supplementary file 1 [file ijms-19-03655-s001.zip › ijms-381771-supplementary/Table S1 Characteristics of TCP genes from cotton..docx]

**Table S2.** Characteristics of TCP genes from cotton. AA: Amino acid; pI: The theoretical isoelectric point of proteins; Mw: The theoretical molecular weight of proteins.

| **Gene name** | **Gene ID** | **CDS(bp)** | | **Deduced polypeptide** | | |
| --- | --- | --- | --- | --- | --- | --- |
|  |  |  |  | **No. of AA** | **pI** | **Mw(kDa)** |
| GhTCP1 | Gh_A13G0528 | | 768 | 255 | 9.66 | 26.37 |
| GhTCP2 | Gh_A01G0414 | | 1341 | 446 | 6.47 | 48.35 |
| GhTCP3 | Gh_A12G2405 | | 1104 | 367 | 8.93 | 41.56 |
| GhTCP4 | Gh_A13G0648 | | 1101 | 366 | 9.44 | 39.72 |
| GhTCP5 | Gh_D01G0419 | | 1335 | 444 | 6.46 | 48.12 |
| GhTCP6 | Gh_D01G1783 | | 1647 | 548 | 6.72 | 57.78 |
| GhTCP7 | Gh_A11G0279 | | 1188 | 395 | 6.91 | 42.27 |
| GhTCP8 | Gh_D12G1689 | | 1506 | 501 | 6.69 | 55.94 |
| GhTCP9 | Gh_D12G1814 | | 984 | 327 | 5.80 | 36.32 |
| GhTCP10 | Gh_A01G1534 | | 1653 | 550 | 6.63 | 57.89 |
| GhTCP11 | Gh_A03G1464 | | 777 | 258 | 9.72 | 26.67 |
| GhTCP12 | Gh_A04G0316 | | 1206 | 401 | 6.34 | 43.74 |
| GhTCP13 | Gh_D04G0925 | | 1311 | 436 | 6.07 | 48.37 |
| GhTCP14 | Gh_A12G2051 | | 1155 | 384 | 8.95 | 41.13 |
| GhTCP15 | Gh_A12G1522 | | 1035 | 344 | 8.53 | 37.56 |
| GhTCP16 | Gh_D04G1732 | | 1464 | 487 | 7.37 | 50.997 |
| GhTCP17 | Gh_D12G1425 | | 891 | 296 | 9.67 | 31.56 |
| GhTCP18 | Gh_D05G2610 | | 1266 | 421 | 6.90 | 44.76 |
| GhTCP19 | Gh_A04G0489 | | 4218 | 405 | 6.47 | 45.19 |
| GhTCP20 | Gh_A04G1120 | | 1464 | 487 | 7.38 | 51.03 |
| GhTCP21 | Gh_A05G1236 | | 1233 | 410 | 6.74 | 44.94 |
| GhTCP22 | Gh_D07G0639 | | 1230 | 409 | 8.48 | 43.11 |
| GhTCP23 | Gh_D07G0680 | | 801 | 266 | 7.78 | 30.33 |
| GhTCP24 | Gh_A05G2343 | | 1269 | 422 | 6.72 | 44.81 |
| GhTCP25 | Gh_A05G2936 | | 903 | 300 | 7.94 | 32.02 |
| GhTCP26 | Gh_A07G0613 | | 801 | 266 | 7.78 | 30.28 |
| GhTCP27 | Gh_A07G1572 | | 1233 | 410 | 9.36 | 45.22 |
| GhTCP28 | Gh_A09G1389 | | 603 | 200 | 8.32 | 21.72 |
| GhTCP29 | Gh_A09G1605 | | 1026 | 341 | 6.26 | 36.93 |
| GhTCP30 | Gh_A09G2496 | | 897 | 298 | 9.52 | 31.42 |
| GhTCP31 | Gh_A10G0394 | | 1392 | 463 | 7.01 | 50.15 |
| GhTCP32 | Gh_A10G0634 | | 906 | 301 | 6.98 | 32.02 |
| GhTCP33 | Gh_A11G0057 | | 1161 | 386 | 9.31 | 44.54 |
| GhTCP34 | Gh_D12G1742 | | 1203 | 400 | 6.52 | 43.29 |
| GhTCP35 | Gh_D12G2229 | | 1158 | 385 | 8.96 | 41.10 |
| GhTCP36 | Gh_D12G2641 | | 1086 | 361 | 8.20 | 40.81 |
| GhTCP37 | Gh_D13G0602 | | 771 | 256 | 9.60 | 26.53 |
| GhTCP38 | Gh_A01G1471 | | 225 | 74 | 10.07 | 8.09 |
| GhTCP39 | Gh_A11G0759 | | 1017 | 338 | 9.08 | 35.41 |
| GhTCP40 | Gh_A12G1214 | | 732 | 243 | 9.90 | 25.32 |
| GhTCP41 | Gh_D13G2530 | | 1098 | 365 | 9.26 | 39.70 |
| GhTCP42 | Gh_Sca026339G01 | | 675 | 225 | 7.91 | 23.63 |
| GhTCP43 | Gh_A12G1561 | | 1506 | 501 | 7.02 | 55.88 |
| GhTCP44 | Gh_A12G1302 | | 891 | 296 | 9.67 | 31.50 |
| GhTCP45 | Gh_A13G0647 | | 1053 | 350 | 9.54 | 38.04 |
| GhTCP46 | Gh_D11G0887 | | 1017 | 338 | 9.08 | 35.50 |
| GhTCP47 | Gh_D12G1337 | | 732 | 243 | 9.99 | 25.37 |
| GhTCP48 | Gh_A13G1272 | | 1230 | 409 | 6.78 | 44.27 |
| GhTCP49 | Gh_A13G2021 | | 591 | 196 | 8.91 | 21.11 |
| GhTCP50 | Gh_D02G1925 | | 774 | 257 | 9.51 | 26.65 |
| GhTCP51 | Gh_D04G0387 | | 942 | 313 | 8.58 | 34.65 |
| GhTCP52 | Gh_D04G0721 | | 903 | 300 | 8.67 | 31.76 |
| GhTCP53 | Gh_D05G3332 | | 1206 | 401 | 6.23 | 43.86 |
| GhTCP54 | Gh_D05G3838 | | 1233 | 410 | 8.76 | 45.06 |
| GhTCP55 | Gh_D07G2330 | | 915 | 304 | 8.65 | 32.51 |
| GhTCP56 | Gh_D08G1913 | | 969 | 322 | 8.42 | 33.72 |
| GhTCP57 | Gh_D09G0381 | | 897 | 298 | 9.49 | 31.46 |
| GhTCP58 | Gh_A12G1603 | | 1221 | 406 | 6.88 | 44.08 |
| GhTCP59 | Gh_A12G1657 | | 978 | 325 | 5.80 | 36.03 |
| GhTCP60 | Gh_D09G1394 | | 606 | 201 | 7.71 | 21.85 |
| GhTCP61 | Gh_D09G1703 | | 1014 | 337 | 5.93 | 36.41 |
| GhTCP62 | Gh_D10G0762 | | 903 | 300 | 8.64 | 31.98 |
| GhTCP63 | Gh_A07G2121 | | 840 | 279 | 9.10 | 29.69 |
| GhTCP64 | Gh_A08G1602 | | 939 | 312 | 9.66 | 34.04 |
| GhTCP65 | Gh_A09G0084 | | 858 | 285 | 7.94 | 31.98 |
| GhTCP66 | Gh_D11G0061 | | 987 | 328 | 8.86 | 37.60 |
| GhTCP67 | Gh_D11G0333 | | 1188 | 395 | 7.04 | 42.27 |
| GhTCP68 | Gh_D13G1576 | | 1230 | 409 | 6.78 | 44.21 |
| GhTCP69 | Gh_A05G3219 | | 930 | 309 | 8.71 | 34.25 |
| GhTCP70 | Gh_A07G0574 | | 1257 | 418 | 8.95 | 44.52 |
| GhTCP71 | Gh_D12G1644 | | 1035 | 344 | 8.85 | 37.57 |
| GhTCP72 | Gh_D13G2419 | | 591 | 196 | 8.93 | 21.13 |
| GhTCP73 | Gh_D13G2529 | | 1056 | 351 | 9.21 | 38.09 |
