# Supplementary figures and images for: Identification, Characterization, and Expression Patterns of TCP Genes and microRNA319 in Cotton
Source: Int J Mol Sci. 2018 Nov 20;19(11):3655. doi: 10.3390/ijms19113655 (PMC6274894; doi:10.3390/ijms19113655)

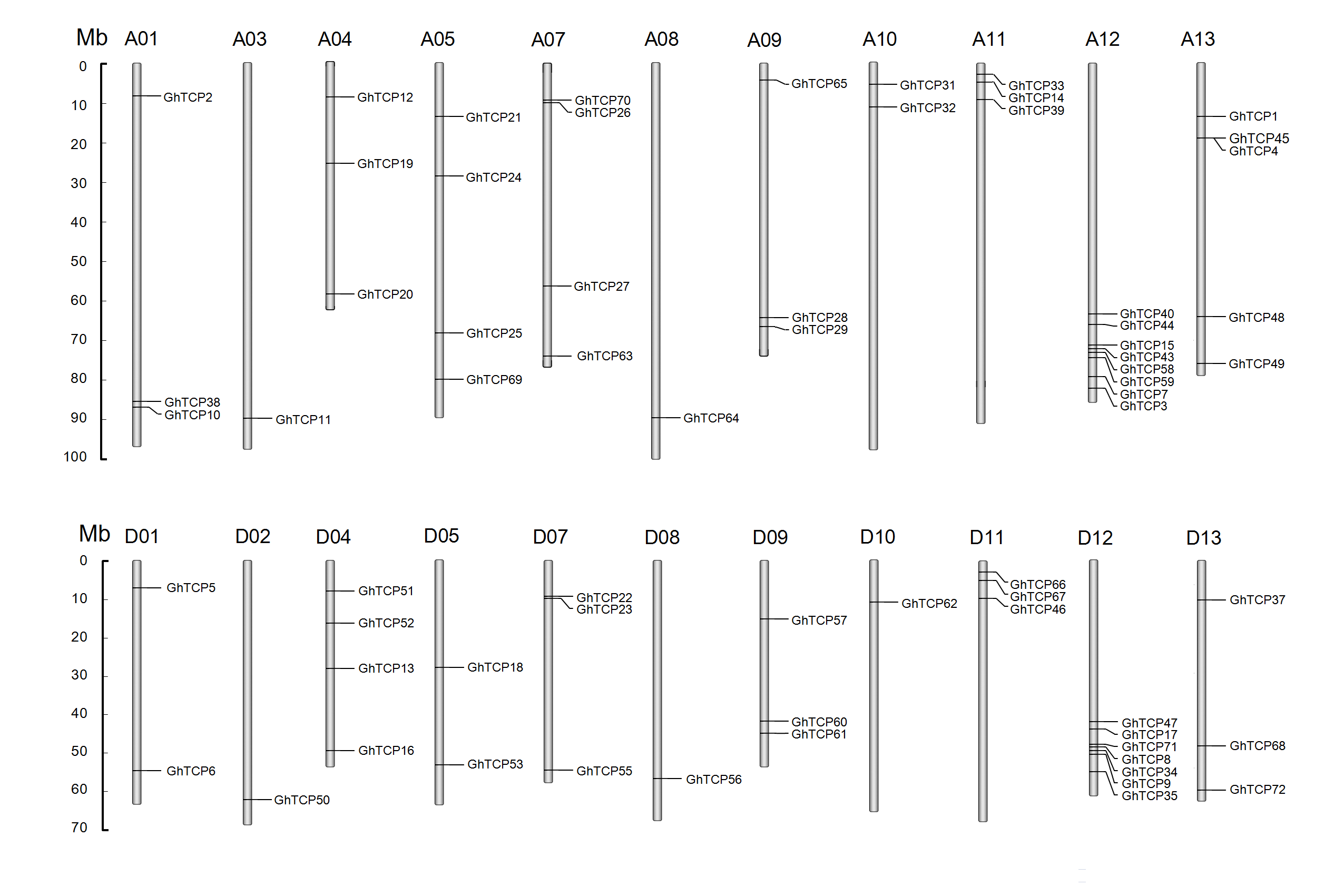

Supplement: Supplementary file 1 [file ijms-19-03655-s001.zip › ijms-381771-supplementary/Figure S1. Locations of TCP genes on G. hirsutum chromosomes.tif]

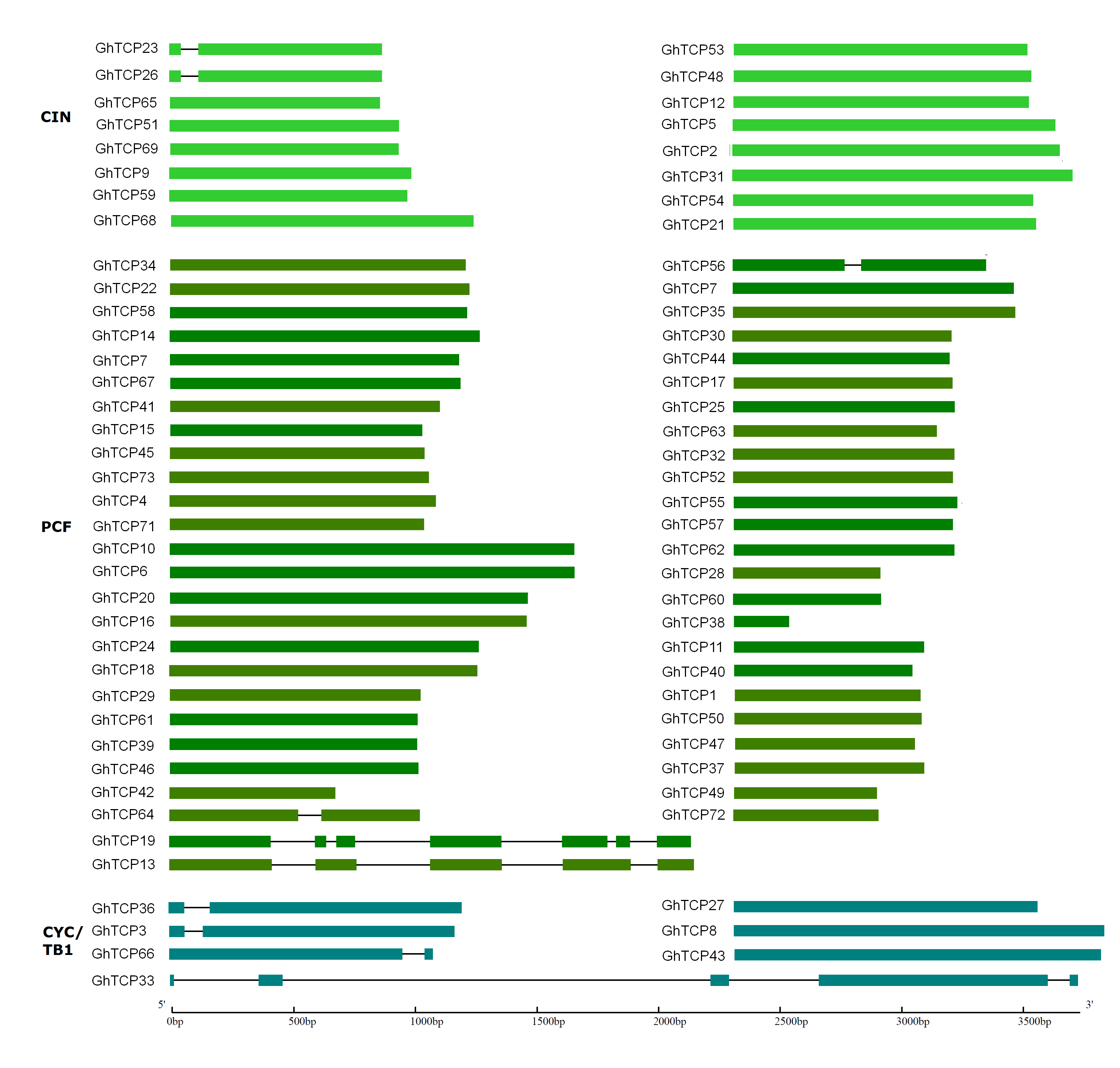

Supplement: Supplementary file 1 [file ijms-19-03655-s001.zip › ijms-381771-supplementary/Figure S2. Structural analysis of G. hirsutum TCP transcription factors.tif]

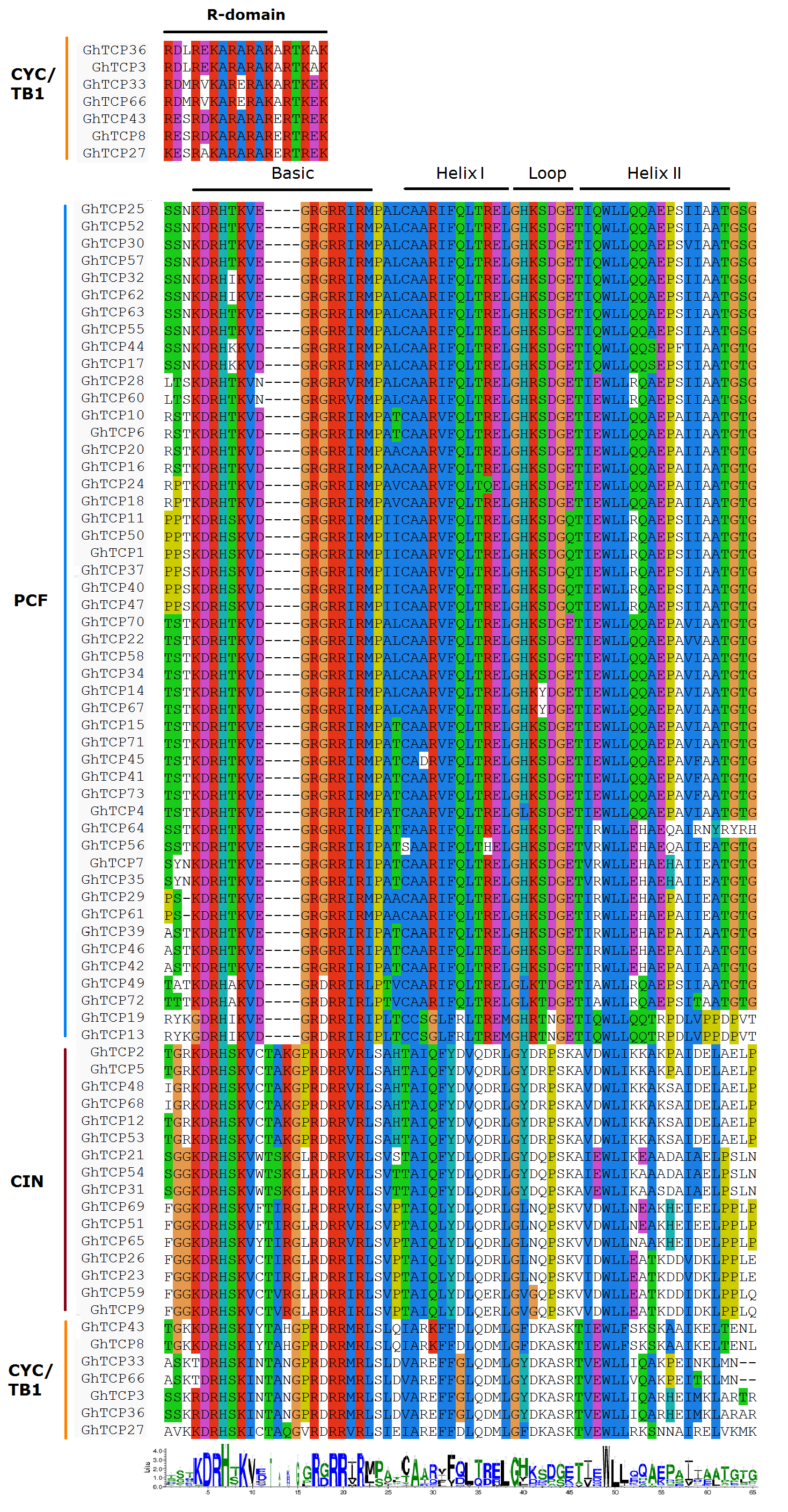

Supplement: Supplementary file 1 [file ijms-19-03655-s001.zip › ijms-381771-supplementary/Figure S3. Multiple sequence alignment of G. hirsutum TCP transcription factors.tif]
